# Relationship Between Body Mass Index, Eating Disorders, Internet Addiction, and Social Media Addictions Among Adolescents in Bangladesh

**DOI:** 10.1002/puh2.70142

**Published:** 2025-10-24

**Authors:** Md. Abu Bakkar Siddik, Shah Jalal Ahmed, Md. Zahid Hasan, Abdulla Al Masud, Md. Yeasin Arafat, Md. Jane Alam, Akher Ali, Muhammd Mustafiz, Md. Al Amin, Mahedi Hasan

**Affiliations:** ^1^ School of the Environment Nanjing University Nanjing China; ^2^ The Center for Social Policy and Justice Dhaka Bangladesh; ^3^ Department of Development Studies Daffodil International University Dhaka Bangladesh; ^4^ Department of Sociology Shahjalal University of Science and Technology Sylhet Bangladesh; ^5^ Department of Communication and Journalism University of Chittagong Chittagong Bangladesh; ^6^ Department of Anthropology Shahjalal University of Science and Technology Sylhet Bangladesh; ^7^ Department of Sociology University of Chittagong Chittagong Bangladesh; ^8^ Department of Political Science University of Chittagong Chittagong Bangladesh; ^9^ Department of Statistics and Data Science Jahangirnagar University Dhaka Bangladesh; ^10^ Department of Radio, Television and Film, Marmara University Istanbul Turkey; ^11^ Department of Agricultural and Applied Economics University of Georgia Athens Georgia USA; ^12^ College of Media and Communication Texas Tech University Lubbock Texas USA

**Keywords:** adolescents, body mass index, eating disorders, internet addiction, social media addiction

## Abstract

**Background:**

Due to the increasing consumption of the internet, several outcomes are often seen among adolescents, including an increase in internet addiction (IA) and social media addiction (SMA). It could also have been associated with high body mass index (BMI) and eating disorders (EDs). This study aimed to assess the relationship between BMI, EDs, IA, and SMA among adolescents in Bangladesh and to determine the associated factors.

**Methodology:**

Using a cross‐sectional design, this study gathered data from adolescents aged 13–19 years old using an online questionnaire consisting of sociodemographic variables, BMI, eating disorder test scale (EAT‐26), Young's Internet addiction test scale (IAT‐20), and Bergen's social media addiction scale (BSMAS). Apart from the descriptive statistics and Pearson chi‐square test, we employed two sets of binary logistic regression and bivariate co‐relation matrix to analyze the data and to find out the relationship by SPSS 26.0.

**Results:**

A total of 2147 adolescents with a mean age of 15.6 years old participated in the study; among them, 70.70% were female. Our study found that 23.2% of participants had EDs, 30.8% of students were addicted to the Internet, and 59.9% of participants were addicted to social media. Concerning BMI, we found that 6.6% of participants were underweight, 1.9% were overweight, 24% were obese, and the remaining 67.5% had a normal BMI. Gender, the purpose of the internet, daily internet use, physical exercise, the study of novels/stories, EDs, and BMI had a significant effect on increasing IA and SMA among adolescents. IA, SMA, and EDs were co‐related.

**Conclusion:**

The stakeholders should inspire and take necessary steps to engage the adolescents in physical exercise and literature habits to control the IA and SMA. And regarding BMI and EDs, the parents should be aware of this.

## Graphical abstract

Adolescents in Bangladesh are more likely to develop internet and social media addiction when male, using the internet for gaming or entertainment, spending long hours online, lacking exercise or reading habits, having eating disorders, or higher BMI. Preventive strategies should target these risk factors.

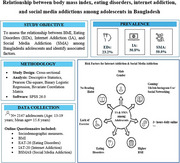

## 1 Introduction

The widespread integration of technologies has brought about significant changes in adolescents’ everyday lives, creating digital spaces that influence their social interactions, behaviors, and lifestyles. With the rise of smartphones, social media platforms, and online entertainment, nearly 90% of the adolescents now depend on digital tools for communication, entertainment, and information [1]. Although this digital immersion offers immense opportunities for connection and learning, it has also introduced significant challenges to adolescent well‐being. The attention of researchers and healthcare professionals has catered to the emerging issues of digital addictions, including internet addiction (IA) and social media addiction (SMA). Research shows that intensive use of the internet and social media has a negative correlation to psychological health, such as anxiety, depression, and social distancing [2, 3, 4]. Furthermore, these digital addictions have been linked to sleep pattern abnormalities, declining academic performance, and difficulties in socialization among adolescents [5, 6, 7]. So, it is imperative to understand the interconnected factors contributing to their health and well‐being in the context of digital advancements.

Body mass index (BMI) is a widely used tool for assessing growth patterns in adolescents. It provides insights into their physical well‐being and potential risks for various health conditions. BMI is a commonly used screening method to identify people who may be at risk of falling into the underweight, normal weight, overweight, or obese categories. It is computed using information from weight and height measurements. Teenagers with high BMI are more likely to experience obesity‐related health problems, such as heart disease, Type 2 diabetes, and musculoskeletal illnesses [8, 9, 10]. On the other hand, people with low BMI may be at risk for malnutrition, weakened immune systems, and stunted growth and development [11]. Furthermore, BMI has also been connected to psychological elements, including teenagers’ low self‐esteem and unsatisfied body image, highlighting its importance in determining general health and well‐being [12, 13]. In addition to BMI, eating disorders (EDs), which are marked by abnormalities in eating patterns and self‐perceptions of one's body, constitute a crucial component of teenage health. A significant percentage of teenagers worldwide suffer from EDs, such as binge ED, bulimia nervosa, and anorexia nervosa, which can have detrimental effects on both physical and mental health [14, 15]. These disorders carry severe consequences, including malnutrition, electrolyte imbalances, gastrointestinal issues, and decreased social functioning [16, 17, 18]. Yet, there is a research gap on the interactions between rising digital dependencies and EDs and BMI, despite the importance of these aspects in adolescent health being increasingly recognized.

Excessive, obsessive, and dysfunctional habits of online and social media consumption characterize the contemporary phenomenon of IA and SMA. IA, also known as problematic internet use or online reliance, is a collection of behaviors that include an inability to control one's internet usage and can have detrimental effects on a person's life in a variety of areas [19, 20, 21]. Signs of IA typically include obsessive internet use, withdrawal feelings while not using the internet, loss of interest in other activities, and prolonged use despite negative effects [22, 23]. Comparably, excessive and uncontrollable usage of social media sites, like Facebook, Instagram, X, and other forms of social media, is referred to as SMA. This can negatively impact interpersonal relationships and daily functioning [24]. Typical diagnostic criteria for SMA include signs like spending too much time on social media, making ineffective attempts to reduce usage, and ignoring other responsibilities because of social media use [25, 26]. Addiction to social media and the internet has gained attention on a global scale, especially among youth, who are particularly drawn to these technologies. Research has indicated varying prevalence rates of internet and SMA in teenagers, contingent upon the characteristics of the group under investigation and the techniques employed for evaluation [27].

The relationships between BMI, EDs, and digital addictions among teenagers are deeply interconnected, with existing literature revealing overlapping vulnerabilities and complex interactions. Research has established links between high BMI indices and teenage vulnerability to digital addictions [28] and observed a higher prevalence of such addictions among adolescents with existing EDs, suggesting common risk factors [29]. More recent studies clarify this connection by identifying problematic social media use (PSMU) as a critical bridge between psychological vulnerabilities and disordered eating behaviors. For example, personality traits like narcissism, a known risk factor for both EDs and exercise addiction, are often channeled through PSMU and exposure to online “fitspiration” content, revealing a clear pathway where personal vulnerability is amplified by online behavior [30, 31]. This digital environment strengthens peer pressure and social comparison [32], which can intensify body dissatisfaction and disordered eating habits [33, 34, 35]. These dynamics are well‐explained by Social Comparison Theory, which posits that adolescents evaluate themselves against idealized online images, intensifying body dissatisfaction that can precipitate disordered eating [36]. Concurrently, Compensatory Internet Use Theory suggests that adolescents may engage in excessive online activity to escape from or cope with real‐life distress, such as negative body image, thereby creating a cycle of problematic addiction [37]. Ultimately, this online preoccupation can manifest as an “overvaluation of shape and weight,” a core cognitive distortion in conditions like binge‐ED [38]. However, there are still a lot of unanswered questions in the field of digital addictions, EDs, and BMI despite mounting evidence to the contrary. Therefore, a comprehensive understanding of these digitally mediated pathways is essential for developing effective, modern interventions to address these interconnected health crises [39].

In this study, we aim to explore the complex links that exist among Bangladeshi adolescents between BMI, EDs, IA, and SMA. The study specifically looks at the relationships between these variables, investigating how adolescents eating and BMI patterns may be impacted by internet usage patterns.

## 2 Methodology

### 2.1 Ethical Approval

The research project was granted logistical assistance and ethical clearance from the Institutional Review Board (IRB) of Noakhali Science and Technology University. The provided reference code was NSTU/SCI/EC/2023/170. Only those participants providing written consent were included in the study; those lacking written consent were not included.

### 2.2 Study Design, Procedure, and Data Collection

The cross‐sectional study comprised 2147 adolescents, aged 13–19, from various selected schools and colleges spread across Bangladesh between March and July of 2023. The research participants’ average age was 15.6 years, along with 70.70% of the participants were female. A simple random sampling method obtained the sample from the specified institutions. At the outset, 67 students filled out a self‐reported questionnaire as part of a pilot survey. After completing the pilot study assessment to evaluate the viability and effectiveness of the research, the entire survey was conducted. A concise online survey application called Google Forms was used to gather data. The questionnaire consisted of sections on sociodemographic factors, BMI, eating disorder test (EAT‐26) scale, internet addiction test (IAT‐20) scale, and Bergen social media addiction scale (BSMAS).

### 2.3 Criteria for Selection

The study's criteria for including participants were as follows: (i) enrollment of students who were currently studying at school; (ii) students who were currently studying at college; (iii) adolescents under the age of 19; and (iv) requirement of Bangladeshi nationality by birth. Exclusion criteria for this research were (i) adolescents with dual citizenship; (ii) foreign students currently studying in Bangladesh; and (iii) adolescents currently studying abroad under the Bangladeshi curriculum.

## 3 Measures

### 3.1 Sociodemographic Measures

Questions regarding sociodemographics were asked, including age, sex (male and female), academic class (8–12), study group (arts, commerce, and science), mother's highest education, father's highest education, main purpose of using internet (chatting, gaming, social networking, watching TikTok or Instagram videos, and others), daily internet usage (1–2, 2–3, 3–4, 4–5, 5–6 h, less than 1 h, more than 6 h), smoking habit (yes and no), reading literature habit (yes and no), and victims of bullying (yes and no).

### 3.2 Body Mass Index (BMI)

The standard formula for BMI is weight in kilograms divided by the square of height in meters (kg/m^2^). We measured height and weight to the closest 1 cm and 0.1 kg. We used the US Centers for Disease Control and Prevention (CDC) age and sex‐specific cut‐off values for BMI. The CDC categorizes BMI into four groups: underweight (<fifth percentile), normal weight (fifth to <85th percentile), overweight (85th to <95th percentile), and obese (≥95th percentile) [40]. We followed the criteria referred by CDC in the current study.

### 3.3 Eating Disorder Questionnaire

The EAT‐26 was used to assess disordered eating behaviors [41]. There were 26 items in the questionnaire, divided into three categories: dieting, bulimia and food preoccupation, and oral control. Responses on a 6‐point scale from “always” to “never” were recorded. For questions 1–25, the responses “always,” “usually,” “often,” “sometimes,” “rarely,” and “never,” were scored as 3, 2, 1, 0, 0, and 0, respectively. The scoring sequence was reversed for question 26. The possible total score ranged from 0 to 78. A score of less than 20 indicates “no risk,” a score of 20–49 indicates “at risk,” and a score of 50–78 is “consistent with an eating disorder” [42]. The scale showed a good consistency with a Cronbach alpha 0.76.

### 3.4 IAT Scale

K. Young created Young's IAT‐20, a unidimensional, standardized psychometric instrument [42]. On the basis of users’ online recreational activities on any internet‐connected device, this validated scale was used to determine the degree of IA. The 20 questions comprising the IAT were scored on a five‐point Likert scale: 0 = Not Applicable, 1 = Rarely, 2 = Occasionally, 3 = Frequently, 4 = Often, and 5 = Always. The examinee's scores for each of the 20 potential responses were added to determine the final score of the IAT. The maximum number of points that may be earned was 100. IA was classified as having a score above 50, whereas not having an addiction was indicated by a score below 50 [42, 43]. The scale showed a good consistency with a Cronbach alpha 0.81.

### 3.5 Social Media Addiction

The BSMAS is a concise and user‐friendly tool created by [44] to evaluate the likelihood of SMA [44], and it has been confirmed as effective for Bangladeshi adolescents [45]. The tool was created using the six fundamental aspects of addiction: salience, mood modulation, tolerance, withdrawal conflict, and relapse. It consists of six items that are assessed on a five‐point Likert scale from 1 (extremely rarely) to 5 (very often). With a total score between 6 and 30, a higher score on the BSMAS indicates an increased likelihood of being addicted to social media, but this study used 24 as a cut‐off score for having SMA among the participants [46]. The scale showed a good consistency with a Cronbach alpha 0.79.

### 3.6 Statistical Analysis

Descriptive analysis included sociodemographic, IA, SMA, EAT, and BMI variables. Score bands on the IAT‐20, BSMAS, and EAT‐26 classified respondents as internet addicted. We used the Pearson chi‐square test to find connections between our parameters and IA and SMA. IA and SMA were the dependent variables in a binary logistic regression model, whereas all other components were independent variables. A Shapiro–Wilk test verified multivariate normality before chi‐square and logistic regression studies. Dataset multicollinearity was examined using a correlation matrix. The Kolmogorov–Smirnov test determined data dependence and confirmed multivariate normality, independence, and absence of multicollinearity. In all categorical variables, odds ratios (OR) and 95% confidence intervals were determined. Pie charts revealed IA, SMA, EAT, and BMI rates among the adolescents. All analyses used SPSS 26.0.

## 4 Results

### 4.1 Description of the Participants

Table 1 shows the descriptive analysis of the study. A total of 2147 students, with a mean age of 15.6 years, participated in the study. The sample was predominantly female, comprising 70.70% (*n* = 1518) of the participants, whereas male students constituted the remaining 29.30% (*n* = 629). Our findings indicated that IA was more frequent among males (45.9%, *n* = 289) than females (24.5%, *n* = 372). In addition, IA was highest among Class 12 students (65.71%, *n* = 69), followed by 48.50% (65) in Class 11, 33% (300) in Class 10, 22.90% (219) in Class 9, and 18.60% (8) in Class 8. In contrast, SMA showed a different pattern, with 65.87% of female students (1000) were addicted to social media compared to 45.63% (287) male students. Additionally, SMA was most common in Class 8 (76.74%, *n* = 33), followed by Class 9 (63.38%, *n* = 606), Class 10 (61.27%, *n* = 557), Class 11 (41.78%, *n* = 56), and Class 12 (33.33%, *n* = 35).

**TABLE 1:** Sociodemographic information of the participants.

		Internet addiction					Social media addiction				
		No		Yes			No		Yes		
Variables		Table total *N* (%)	Count	Table total *N* (%)	Count	*p* value	Table total *N* (%)	Count	Table total *N* (%)	Count	*p* value
Age	Mean age 15.6 years old										
Gender	Female	53.4	1146	17.3	372		24.1	518	46.6	1000	
	Male	15.8	340	13.5	289		15.9	342	13.4	287	
Class	Class 12	1.7	36	3.2	69		3.3	70	1.6	35	
	Class 8	1.6	35	0.4	8		0.5	10	1.5	33	
	Class 10	28.4	609	14.0	300		16.4	352	25.9	557	
	Class 11	3.2	69	3.0	65		3.6	78	2.6	56	
	Class 9	34.3	737	10.2	219		16.3	350	28.2	606	
Group	Arts	30.4	652	11.7	252		16.6	356	25.5	548	
	Commerce	4.1	87	3.4	72		3.4	74	4.0	85	
	Science	34.8	747	15.7	337		20.0	430	30.5	654	
Mothers highest education	I do not know	14.1	303	6.7	143		8.8	190	11.9	256	
	University	10.2	218	5.0	107		6.3	135	8.8	190	
	Up to college level	8.9	192	6.9	148		6.4	137	9.5	203	
	Up to school level	36.0	773	12.2	263		18.5	398	29.7	638	
Father's highest education	I do not know	15.1	324	6.7	144		9.1	195	12.7	273	
	University	14.9	319	8.1	174		8.6	185	14.3	308	
	Up to college level	10.8	232	7.2	155		8.5	182	9.5	205	
	Up to school level	28.5	611	8.8	188		13.9	298	23.3	501	
Father's occupation	Businessmen	26.0	558	14.8	317		17.7	379	23.1	496	
	Farmer	5.4	117	1.1	23		2.2	47	4.3	93	
	Job holder	16.3	350	7.5	160		8.7	186	15.1	324	
	Others	21.5	461	7.5	161		11.6	248	17.4	374	
Purpose of internet use?	Chatting	0.7	15	2.6	56		2.1	46	1.2	25	
	Gaming	2.6	55	1.4	29		1.4	31	2.5	53	
	Others	4.6	99	3.7	79		3.8	81	4.5	97	
	Social networking	26.8	575	17.9	384		22.9	492	21.8	467	
	Study	34.6	742	5.3	113		9.8	210	30.0	645	
Daily internet usage	1–2 h	18.1	389	5.7	122		8.5	183	15.3	328	
	2–3 h	5.3	114	5.8	125		7.1	153	4.0	86	
	3–4 h	3.1	67	4.2	90		4.0	86	3.3	71	
	4–5 h	1.0	21	3.1	67		3.2	68	0.9	20	
	5–6 h	0.7	16	1.4	30		1.9	41	0.2	5	
	Less than 1 h	40.5	870	6.5	140		11.3	242	35.8	768	
	More than 6 h	0.4	9	4.1	87		4.1	87	0.4	9	
Smoke habit	No	67.9	1457	29.3	628		38.1	817	59.1	1268	
	Yes	1.4	29	1.5	33		2.0	43	0.9	19	
Reading habit	No	26.5	569	15.0	322		18.4	395	23.1	496	
	Yes	42.7	917	15.8	339		21.7	465	36.8	791	
Eating disorder	No	58.9	1265	17.9	384		26.2	562	50.6	1087	
	Yes	10.3	221	12.9	277		13.9	298	9.3	200	
Body mass index	<18	4.1	88	2.5	53		3.1	67	3.4	74	
	>30	15.5	332	8.6	184		10.3	222	13.7	294	
	18–24	48.3	1037	19.2	413		25.9	556	41.6	894	
	25–30	1.4	29	0.5	11		0.7	15	1.2	25	

### 4.2 Prevalence of Eating Disorders, Internet Addiction, Social Media Addiction, and Body Mass Index Categories

**FIGURE 1:**
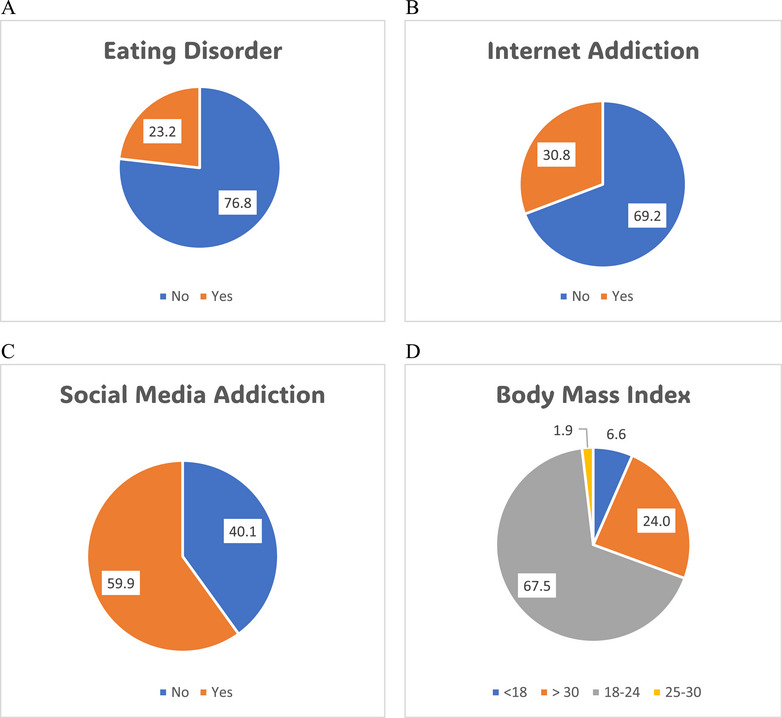
Different levels of (D) body mass index and prevalence of (A) eating disorders, (B) internet addiction, and (C) social media addiction among adolescents.

Figure 1 demonstrates the prevalence of EDs (Figure 1A), IA (Figure 1B), SMA (Figure 1C), and different categories of BMI (Figure 1D) among the participants. The findings revealed that 23.2% of respondents reported having EDs, whereas the majority (76.8%) did not. Additionally, IA was present in 30.8% of adolescents, with 69.2% unaffected. Furthermore, 59.9% of participants were addicted to social media, with the remaining 40.1% not exhibiting SMA. Concerning BMI, we found that 6.6% of participants were underweight, 1.9% were overweight, 24% were obese, and the remaining 67.5% had a normal BMI.

### 4.3 Factors Associated With Internet Addiction and Social Media Addiction

Binary logistic regression analysis (Table 2) identified several factors significantly associated with IA and SMA among adolescents in Bangladesh, including gender (*p* = 0.002 for IA; *p* < 0.001 for SMA), the purpose of the internet use (*p* < 0.001 for all), daily internet use (*p* < 0.001), physical exercise (*p* < 0.001 for IA; *p* = 0.002 for SMA), the study of novels/stories (*p* < 0.001), EDs (*p* < 0.001), and BMI (*p* < 0.001 for IA; *p* = 0.009 for SMA).

**TABLE 2:** Binary logistic regression analysis results.

		Internet addiction		Social media addiction	
Variables	Categories	Exp(*B*) (lower–upper)	*p* value	Exp(*B*) (lower–upper)	*p* value
Age					
Gender	Male	Reference	0.002	Reference	<0.001
	Female	0.56 (0.24–1.31)		0.48 (0.29–1.16)	
Purpose of using internet	Studying	Reference	<0.001	Reference	<0.001
	Gaming	2.04 (1.86–3.02)		2.38 (2.08–2.79)	
	Others	1.82 (1.4–2.36)		1.71 (1.18–2.13)	
	Social networking	3.14 (2.78–3.90)		3.60 (3.20–3.84)	
	Watching TikTok, Instagram videos	9.74 (9.17–10.19)		4.26 (3.88–4.87)	
Daily internet use (h)	<1	Reference	<0.001	Reference	<0.001
	1–2	0.13 (.03–0.70)		0.73 (0.55–2.58)	
	2–3	0.21 (0.02–0.53)		0.85 (0.65–2.65)	
	3–4	1.2 (1.02–1.76)		5.06 (4.81–5.41)	
	4–5	2.8 (2.01–2.99)		3.02 (2.52–4.51)	
	5–6	3.5 (3.07–3.80)		4.54 (4.25–5.11)	
	>6	5.5 (5.01–6.27)		5 (4.54–5.24)	
Physical exercise	Yes	Reference	<0.001	Reference	0.002
	No	3.8 (3.09–4.10)		1.09 (.34–3.51)	
Reading habit	No	Reference	<0.001	Reference	<0.001
	Yes	0.45 (0.31–1.40)		0.46 (0.26–1.05)	
Eating disorder	No	Reference	<0.001	Reference	<0.001
	Yes	1.69 (1.45–2.29)		2.82 (2.45–2.95)	
BMI	<18	Reference	<0.001	Reference	0.009
	18–24	1.46 (1.37–1.77)		1.56 (1.24–1.88)	
	25–30	1.52 (1.44–1.88)		1.74 (1.55–1.92)	
	>30	2.64 (2.08–3.31)		2.31 (1.82–3.43)	

Abbreviation: BMI, body mass index.

Table 2 further shows the factors behind the development of IA and SMA among adolescents in Bangladesh. We found that males were (1/0.56) = 1.79 times [OR = 0.56, 95% CI = 0.24–1.31] more likely to develop IA and (1/0.48) = 2.08 times [OR = 0.48, 95% CI = 0.29–1.16] more likely to develop SMA compared to female students.

The purpose of internet use emerged as a strong predictor. Compared to students who primarily used internet or study, those using it for gaming purposes were 2.04 times [OR = 2.04, 95% CI = 1.86–3.02] and 2.38 times [OR = 2.38, 95% CI = 2.08–2.79] increased risk of getting addicted to internet and social media, respectively. Similarly, adolescents engaged in social networking showed increased risks of IA [OR = 3.14, 95% CI = 2.78–3.90] and SMA [OR = 3.60, 95% CI = 3.20–3.84]. The highest risks were observed among those who used internet for watching TikTok, Instagram videos, which elevated the odds of IA by 9.74 times [OR = 9.74, 95% CI = 9.17–10.19] and SMA by 4.26 times [OR = 4.26, 95% CI = 3.88–4.87]. Other purposes of internet use were also associated with elevated risks of IA [OR = 1.82, 95% CI = 1.4–2.36] SMA [OR = 1.71, 95% CI = 1.18–2.13] compared to studying.

Daily internet use showed a dose‐response association with both addictions. For IA, using internet 1–2 h [OR = 0.13, 95% CI = 0.03–0.70] and 2–3 h/day [OR = 0.21, 95% CI = 0.02–0.53], was linked to lower odds of getting addicted to IA, but the risk rose steadily with longer use, 3–4 h [OR = 1.20, 95% CI = 1.02–1.76], 4–5 h [OR = 2.80, 95% CI = 2.01–2.99], 5–6 h [OR = 3.50, 95% CI = 3.07–3.80], and >6 h [OR = 5.50, 95% CI = 5.01–6.27]. For SMA, risk escalated sharp from 3 to 4 h of daily use [OR = 5.06, 95% CI = 4.81–5.41] and remained consistently high at 4–5 h [OR = 3.02, 95% CI = 2.52–4.51], 5–6 h [OR = 4.54, 95% CI = 4.25–5.11], and more than 6 h [OR = 5, 95% CI = 4.54–5.24] compared to students who did not use the internet for an hour per day.

Students who did not exercise regularly were 3.8 times [OR = 3.8, 95% CI = 3.09–4.10] more internet addicted and 1.09 times [OR = 1.09, 95% CI = 0.34–3.51] more social media addicted than others who exercised regularly. Similarly, those without a reading habit were (1/0.45) = 2.22 times [OR = 0.45, 95% CI = 0.31–1.40] more likely to develop IA and (1/0.46) = 2.17 times [OA = 0.46, 95% CI = 0.26–1.05] increased risk of SMA than students who studied novels/stories.

As shown in Table 2, students at risk of an ED were 1.69 times [OR = 1.69, 95% CI = 1.45–2.29] more internet addicted and 2.82 times [OR = 2.82, 95% CI = 2.45–2.95] more social media addicted than others who were not at risk of an ED. In terms of BMI, we found that students with normal weight were 1.46 times [OR = 1.46, 95% CI = 1.37–1.77]; overweight were 1.52 times [OR = 1.52, 95% CI = 1.44–1.88]; and obese were 2.64 times [OR = 2.64, 95% CI = 2.08–3.31] more likely to exhibit IA than others with underweight. Additionally, compared to their underweight peers, students with normal weight were 1.56 times [OR = 1.56, 95% CI = 1.24–1.88]; overweight were 1.74 times [OR = 1.74, 95% CI = 1.55–1.92]; and obese were 2.31 times [OR = 2.31, 95% CI = 1.82–3.43] more likely to be addicted to social media.

### 4.4 Co‐Relation Between Internet Addiction, Social Media Addiction, and Eating Disorders

Table 3 presents substantial correlations between gender, IA, SMA, and Beds. Gender, as a binary variable, showed a significant correlation with IA (*r* = 0.632 and *p* < 0.01), SMA (*r* = 0.590 and *p* < 0.01), and EDs (*r* = 0.513 and *p* < 0.01). IA is strongly correlated with SMA (*r* = 0.854 and *p* < 0.01) and EDs (*r* = 0.684 and *p* < 0.01). SMA also shows a strong positive correlation with EDs (*r* = 0.871 and *p* < 0.01).

**TABLE 3:** Bivariate correlation analysis.

Variables	1	2	3	4
Gender	1			
Internet addiction	0.632*	1		
Social media addiction	0.590*	0.854*	1	
Eating disorders	0.513*	0.684*	0.871*	1

*Note:* Gender is a dummy variable.

* When *p*‐valus is less than 0.01.

## 5 Discussion

The rapid digitization of societies worldwide has brought transformative changes in adolescent lifestyles, socialization, and health behaviors. Although digital technologies provide vast opportunities for education, communication, and entertainment, they also pose substantial risks, including behavioral addictions and disordered eating. Adolescents are particularly vulnerable, given their developmental stage and heightened susceptibility to peer influence, identity formation, and online pressures [47]. Emerging evidence suggests that IA and SMA are growing public health concerns in low‐ and middle‐income countries, where digital access is expanding rapidly but psychosocial safeguards may be limited [48]. Against this backdrop, our study examined the relationship between BMI, EDs, IA, and SMA, as well as the factors associated with these conditions among Bangladeshi adolescents.

Our study revealed concerning prevalence rates, with 23.2% of adolescents identified as being at risk for EDs, 30.8% exhibiting IA, and a striking 59.9% showing signs of SMA. This trend is not surprising given that the last decade saw a nearly sixfold increase in internet usage worldwide, bringing about 40% of the world's population online [49]. However, the prevalence of IA varies significantly across regions; for example, rates among adolescents in Europe and the USA have been reported between 7.9% and 25.2%, whereas studies in Asia show a much higher and wider variation, ranging from 8.1% to 50.9% [50]. This issue is particularly intensified in Bangladesh, where extensive government efforts to digitize the nation have led to unparalleled internet accessibility. By February 2018, the number of internet subscribers had already reached over 83 million, covering about half the population, with mobile users constituting the vast majority at over 77 million [51]. This widespread availability of internet access is a likely contributor to the high addiction rates observed. Specifically, the 59.9% prevalence of SMA observed in our study is significantly higher than the 29.4%–37.1% range reported in previous Bangladeshi studies focused on Facebook addiction [52, 53, 54]. This discrepancy may stem from our use of a broader scale compared to the Bergen Facebook Addiction Scale (BFAS) used in prior research. Furthermore, the societal shifts during the COVID‐19 pandemic, where the internet and social media became essential tools for education and social connection during lockdowns, likely accelerated these addictive behaviors among adolescents [55].

Several factors emerged as significant predictors of IA and SMA. Consistent with previous findings in Bangladesh, males demonstrated higher rates of both internet and SMA than females [56, 57]. This gender disparity is often attributed to the tendency of males engaging more in activities like online gaming, gambling, pornography consumption, and exploratory online activities [58, 59]. However, the literature presents a mixed picture, with other studies finding no significant gender differences or even higher rates of SMA in females [60, 61, 62]. These conflicting findings suggest that gender's role in IA is not universal but is heavily shaped by specific cultural contexts. For example, social restrictions that limit outdoor recreational activities for adolescent girls in Bangladesh may lead them to seek social engagement online, potentially increasing their risk in ways not seen in other cultures [63, 64].

Additionally, the duration and purpose of internet use were strongly correlated with addiction. Our findings indicated that adolescents who used internet for more than 3 h/day and using it for nonacademic purposes, such as gaming, social networking, and watching short‐form videos on platforms like TikTok, were significantly more likely to be addicted than those using the internet primarily for studying and spending less than 1 h. This aligns with previous research demonstrating a clear link between time spent on social media and addiction severity [65, 66]. The appeal of free and constant entertainment, the use of the internet as a tool for stress alleviation, the addictive design of apps such as TikTok and Instagram reels, and the formation of identity within online communities all contribute to this vulnerability [67, 68, 69]. For some, particularly those feeling disconnected from their families, the internet and social media can become a coping strategy for negative emotions like depression, further driving excessive use [70, 71].

The present study also found that adolescents who did not engage in regular physical exercise had higher rates of IA and SMA. This finding aligns with and supports a consistent pattern observed in previous research within Bangladesh, where multiple studies have demonstrated that individuals who do not exercise regularly report higher levels of internet and social media dependency [60, 61, 72, 73]. The underlying reason for this connection may be psychological. Studies suggest that a lack of physical exercise can contribute to increased stress and negative emotions, which prompts individuals to turn to the internet and social media as a coping strategy for a sedentary lifestyle [74]. This behavior is part of a broader, often cyclical, pattern where IA is linked to more uncontrolled and unhealthy life choices, and vice versa [75]. Interestingly, the study also showed that adolescents who did not read novels or stories were more addicted to the internet and social media. This finding is different from an Egyptian study that found no such connection. The difference could be due to cultural differences or the unique context of the COVID‐19 pandemic during which that study was conducted [76, 77]. However, recent research from Turkey corroborates the present study's finding, suggesting that engagement with literature may serve as a protective factor against digital addictions [78].

Our findings indicate that the risk of EDs was significantly associated with both IA and SMA. The prevalence of these addictions is higher among adolescents at risk for an ED (23.2%), echoing findings from India, China, and Turkey [29, 79]. This trend can be attributed to broad cultural transitions across the Asia‐Pacific regions, including economic expansion, urbanization, and fundamental shifts in gender roles, family life, and nutritional practices [80, 81, 82]. This phenomenon is particularly evident in Bangladesh, which is undergoing a nutritional and cultural transition. This shift is characterized by rising fast‐food consumption, the economic transition, and greater exposure to Western beauty standards through digital media, likely contributing to the higher prevalence of EDs [83, 84, 85]. Social media platforms can intensify the risk of EDs by promoting unrealistic thinness ideals, whereas the internet facilitates unhealthy eating patterns through easy access to high‐calorie food delivery services [86, 87, 88]. This digital exposure, combined with a potential lack of parental monitoring, creates a vulnerable environment where disordered eating habits can develop [89, 90, 91]. Not surprisingly, we found that adolescents at risk of EDs also reported higher levels of BMI. The result showed that adolescents classified as underweight, overweight, or obese were more addicted to the internet and social media than their normal‐weight peers. Similar results were obtained from previous studies conducted among adolescents in Bangladesh [92, 93]. This is due to the fact that for overweight or obese individuals facing social stigmatization and isolation, the virtual world may offer a refuge by providing a space for interaction where they feel accepted and not judged by their physical appearance [94].

To theorize the underlying pathways of these observed associations, we can apply the frameworks of Social Comparison Theory and Compensatory Internet Use Theory to distinguish between potential causal variables [36, 37]. On the basis of our findings, certain characteristics may function as predisposing variables that increase an adolescent's initial vulnerability. Specifically, having a non‐normal BMI (underweight, overweight, or obese) may predispose individuals to seek refuge online from offline social stigmatization, a notion supported by the Compensatory Internet Use Theory. The subsequent process of using the internet and social media to cope with this distress then becomes a primary mediating pathway toward addiction. Once addiction develops, a second mediating process, explained by Social Comparison Theory, is activated. Through excessive exposure to curated online content, adolescents might engage in frequent upward social comparisons with idealized body types. This repeated exposure fosters body dissatisfaction, a critical psychological mediator that directly precedes disordered eating behaviors. The strength of this entire causal chain appears to be influenced by several moderating variables identified in our study. For example, the duration and the purpose of internet use (>3 h/day for nonacademic purposes) strengthens exposure to idealized content, thereby intensifying the negative effects of social comparison. Concurrently, the lack of engagement in protective offline activities, such as regular physical exercise or reading novels, may function as a moderator by removing healthy adaptive strategies, thus strengthening the reliance on compensatory internet use.

This study's findings indicate that BMI, EDs, IA, and SMA are not separate challenges but are part of an interconnected web of health issues affecting Bangladeshi adolescents. The findings emphasize an urgent need for comprehensive, integrated interventions and public health strategies that address these factors holistically to support the well‐being of the younger generation. Further research is needed on the relationship between various cultural factors specific to Bangladesh and these issues.

### 5.1 Strengths and Limitations

To the best of the author's knowledge, this is the first‐ever study in Bangladesh to find out the relationship between IA, SMA, EDs, and BMI the adolescents. The major limitation of this research is the study design. This is a cross‐sectional study that may vary from longitudinal research. Additionally, reliance on self‐reported data, particularly for height and weight used in BMI calculation, may introduce reporting bias and affect the accuracy of prevalence estimates, potentially weakening the observed associations. Moreover, the dichotomization of continuous scale data into binary categories results in a loss of valuable information regarding the severity of symptoms across a spectrum, which may have reduced statistical power and obscured the more detailed relationships between these variables. Despite the disadvantages, the findings represent the primary strengths of this research. Additionally, this research identified the literary habits of teenagers and their correlation with IA and SMA as a significant discovery.

## 6 Conclusion

On the basis of the findings of this study, there is a connection between BMI, EDs, IA, and SMA. IA and SMA among adolescents are significantly influenced by factors such as gender, the reason for using the internet, the length of time spent using the internet each day, physical activity, EDs, and BMI. Furthermore, the results of this study demonstrated that how teenagers approach the study of literature has a discernible and unfavorable connection with both IA and SMA. Exercising regularly and developing a habit of reading literature are two things that teenagers should do to lower their risk of developing IA and SMA. In addition to addressing the issue of high BMI, parents and other stakeholders should take the initiative to regulate the amount of time that teenagers spend on the internet daily as well as to encourage them to participate in activities such as reading, exercising, or spending time outside.

## Author Contributions

Md. Abu Bakkar Siddik: conceptualization, methodology, formal analysis, writing – original draft, writing – review and editing, supervision, project administration, funding acquisition. Shah Jalal Ahmed: data curation, writing – original draft, project administration. Md. Zahid Hasan: data curation, methodology, formal analysis, writing – original draft. Abdulla Al Masud: data curation, methodology, investigation. Md. Yeasin Arafat: data curation, methodology, investigation, visualization. Md. Jane Alam: data curation, software, resources, visualization. Akher Ali: formal analysis, writing – review and editing. Muhammd Mustafiz: software, data curation. Md. Al Amin: validation, resources. Mahedi Hasan: validation, data curation.

## Funding

The authors have nothing to report.

## Conflicts of Interest

The authors declare no conflicts of interest.

## Data Availability

Data will be provided upon reasonable request to the corresponding author.
